# Increased Accuracy of Emotion Recognition in Individuals with Autism-Like Traits after Five Days of Magnetic Stimulations

**DOI:** 10.1155/2020/9857987

**Published:** 2020-07-04

**Authors:** Pingping Liu, Guixian Xiao, Kongliang He, Long Zhang, Xinqi Wu, Dandan Li, Chunyan Zhu, Yanghua Tian, Panpan Hu, Bensheng Qiu, Gong-Jun Ji, Kai Wang

**Affiliations:** ^1^Department of Neurology, The First Affiliated Hospital of Anhui Medical University, Hefei 230000, China; ^2^Anhui Province Key Laboratory of Cognition and Neuropsychiatric Disorders, Hefei 230032, China; ^3^Collaborative Innovation Centre of Neuropsychiatric Disorder and Mental Health, Hefei 230000, China; ^4^The Fourth People's Hospital of Hefei, Hefei 230000, China; ^5^Department of Medical Psychology, Chaohu Clinical Medical College, Anhui Medical University, Hefei 230000, China; ^6^Centers for Biomedical Engineering, University of Science and Technology of China, Hefei 230000, China

## Abstract

Individuals with autism-like traits (ALT) belong to a subclinical group with similar social deficits as autism spectrum disorders (ASD). Their main social deficits include atypical eye contact and difficulty in understanding facial expressions, both of which are associated with an abnormality of the right posterior superior temporal sulcus (rpSTS). It is still undetermined whether it is possible to improve the social function of ALT individuals through noninvasive neural modulation. To this end, we randomly assigned ALT individuals into the real (*n* = 16) and sham (*n* = 16) stimulation groups. All subjects received five consecutive days of intermittent theta burst stimulation (iTBS) on the rpSTS. Eye tracking data and functional magnetic resonance imaging (fMRI) data were acquired on the first and sixth days. The real group showed significant improvement in emotion recognition accuracy after iTBS, but the change was not significantly larger than that in the sham group. Resting-state functional connectivity (rsFC) between the rpSTS and the left cerebellum significantly decreased in the real group than the sham group after iTBS. At baseline, rsFC in the left cerebellum was negatively correlated with emotion recognition accuracy. Our findings indicated that iTBS of the rpSTS could improve emotion perception of ALT individuals by modulating associated neural networks. This stimulation protocol could be a vital therapeutic strategy for the treatment of ASD.

## 1. Introduction

Autism spectrum disorders (ASD) are characterized by an early onset of difficulties associated with decreased social communication, restrictive interests, and repetitive patterns of behavior [1, 2]. Obvious social deficits include reduced interest in observing faces and difficulties in understanding facial expressions [3, 4]. Specifically, a number of studies have shown that individuals with ASD are significantly more likely to look at the noncharacteristic areas of the face than the core characteristic areas (such as eyes) [5–7]. ASD patients face serious complications along with the societal and familial challenges, but successful treatment is still elusive. Individuals with autism-like traits (ALT) are a subclinical group with similar social and communication skill deficits as those with ASD. Genetic studies have shown that individuals with ALT and ASD share genetic susceptibility factors [8, 9]. In practice, psychological experiments are much easier to be completed in individuals with ALT than ASD [10]. Thus, ALT has been widely investigated with an ultimate goal to understand ASD's mechanism and to develop potential therapeutic strategies [11, 12].

Faces are multidimensional stimuli bearing important social signals, such as gaze direction and emotional expression [13]. Emotion recognition has a profound influence on social development. Deficits in emotion recognition in ASD have been demonstrated by meta-analysis [14] and can be improved with behavior intervention [15]. Similar deficits were found in individuals with high autism traits as well [2], which may relate to weakened attention responses to eye gazing cue tasks [16, 17]. In humans, the posterior superior temporal sulcus (pSTS) is a cortical locus for processing the dynamic aspects of faces [18]. The pSTS in the right hemisphere plays an important role in social perception [19]. In ASD, a neuroimaging study indicated that the right pSTS was abnormally activated during emotion perception [20]. These findings support the neurobiological theories that emphasize the importance of the STS in facial emotion recognition in ASD [21]. They also implicate the right pSTS as a potential target for modulating the emotion recognition ability of ALT individuals.

With the popularity of noninvasive brain stimulation, repetitive transcranial magnetic stimulation (rTMS) has received much attention in recent years. It has shown potential clinical benefits in several psychiatric disorders, including major depressive disorder [22], schizophrenia [23], and ASD [24]. TMS might prove an effective treatment tool to improve social, cognitive, and emotional communications [25, 26]. Theta burst stimulation (TBS) is a relatively novel protocol of rTMS. Two patterns of TBS, including continuous (c) and intermittent (i), can inhibit and promote cortical excitability, respectively [27]. By cTBS of the rpSTS, eye fixation time was significantly decreased in healthy subjects [28]. Some studies focused on the short-term aftereffects of TMS on social cognition [29–31], but few have investigated the cumulative aftereffects of long-term (days) iTBS on ASD. Such long-term aftereffects may be more easily generalized to clinical applications.

In this study, we hypothesized that an excitatory rTMS protocol would improve emotion recognition in ALT individuals. To this end, ALT individuals were randomly assigned to the active and sham groups receiving iTBS of the pSTS for five consecutive days. Eye tracking experiments were used to show the aftereffects on facial emotion recognition. Functional magnetic resonance imaging (fMRI) was used to show the neural correlates underlying the behavior effect.

## 2. Materials and Methods

### 2.1. Participants

To recruit ALT individuals for this study, we estimated the autism traits of 1000 undergraduates from Anhui Medical University using the autism spectrum quotient (AQ). AQ is a commonly used quantitative measurement method of ASD. It is composed of 50 questions in total and includes five self-rating scales, including social skills, verbal communication, attention to detail, attention shifting, and imagination, covering the main clinical manifestations and behavioral characteristics of autism. The scoring range is from 50 to 200. The higher the score, the more obvious are the autistic characteristics. We defined subjects with AQ > 124 (the top 10% of the total number) as having ALT. One hundred ALT students were subsequently invited to participate in this rTMS study, but only 32 agreed (13 men and 19 women, aged between 21 and 24). All participants were right-handed and had normal vision, hearing, and speech skills. No participant had a history of head trauma, epilepsy, or mental illness or had participated in other TMS studies before.

All subjects underwent a complete set of neuropsychological background tests, including the Montreal Cognitive Assessment Test (MoCA), the Hamilton Depression Rating Scale (HAMD), the Hamilton Anxiety Rating Scale (HAMA), the Stroop test (color, word, and interference tests), the Trail Making Test (A and B), and the Digit Span assessment (forward, backward). The study was approved by the Ethics Committee of Anhui Medical University, and all participants in the behavioral experiment signed an informed consent form.

### 2.2. Study Design

**Figure 1:**
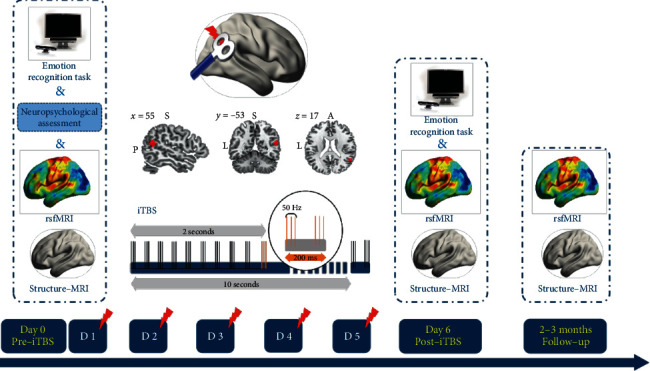
Schematic of the primary experiment. Each subject received real or sham iTBS for five consecutive days (red lightning symbol represents iTBS). The stimulus target, the rpSTS, was defined as a sphere with a center radius of 6 mm based on the Montreal Neurological Institute (MNI) coordinates [55, -53, 17]. Eye tracking and multiple MRI data were acquired before and after the 5-day stimulation. In addition, neuropsychology tests and MRI data were acquired at baseline and 2-3 months after the end of the experiment, respectively.

This double-blinded trial randomly divided subjects with ALT into two groups, receiving either real or sham stimulation. All subjects received eye tracker tests and fMRI scans before the first rTMS intervention. After five consecutive days of rTMS, the eye tracking experiment and fMRI scanning were performed on the sixth experimental day. Two to three months later, resting-state functional and structural images were collected to show the follow-up changes (Figure 1). To assess the integrity of blinding, subjects were asked to guess which intervention they had received at the end of five days of stimulation.

### 2.3. Eye Tracking Experiment

#### 2.3.1. Apparatus and Stimuli

The iView XIII Hi-speed eye tracking device from SMI (SensoMotoric Instruments, Germany) was used to measure eye tracking. The screen resolution of the display was 1280 × 1024 px. Eye movement and pupil size were recorded at a rate of 60 data points per second (60 Hz), and the system tracked both eyes separately by emitting infrared light to create a corneal reflection. The eye tracking cameras and the infrared light source were mounted beneath the screen monitor and did not require fixing of the participants' heads. The distance between the center of the stimulus display screen and the eyes of the subject was about 60 cm [32]. The stimulus photographs for this experiment were from the Chinese Affective Picture System (CAPS) [33]. The experiment began with a nine-point calibration routine that was further verified by four points displayed on the screen. Recalibration is required when drift exceeded 1 degree. Analysis of eye movements and coding of fixation data were performed with BeGaze Analyzing Software (Gaze Intelligence, Paris, France).

#### 2.3.2. Emotion Recognition Task

**Figure 2:**
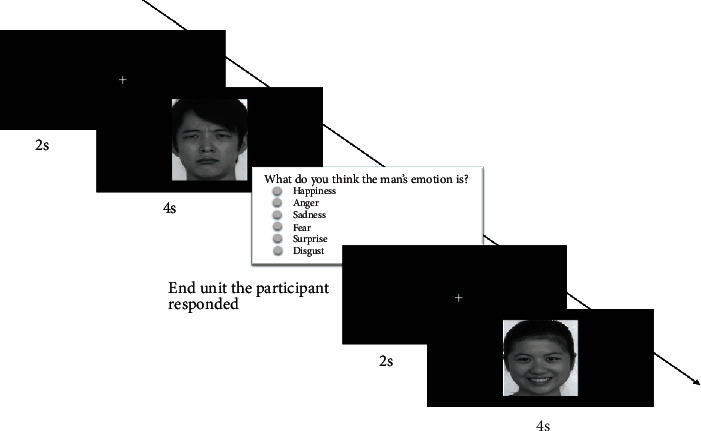
Schematic overview of the emotion recognition task. Each trial started with a fixation cross on a black background for two seconds; then, one of the 36 facial images was displayed for four seconds. Each trial ended with a question and six answer options, which were displayed until the participant responded. Participants were required to verbally provide an answer to each question (e.g., “The emotion of the characters in the picture is: happy.”). Participants were instructed to identify the emotions portrayed in each facial image. Each participant completed 36 trials in total. In the experiment, the questions and six answer options were shown in Chinese.

In the emotion recognition task, participants were randomly shown 36 images of faces from six subjects (two genders by three ages). Each image showed one of the six basic emotions: happiness, anger, sadness, fear, surprise, or disgust. Each trial started with a fixation cross on a black background for two seconds; then, one of the 36 face images was displayed for four seconds. Each trial ended with a question and six answer options, which were displayed until the participant responded. Participants were required to verbally provide an answer to each question (e.g., “The emotion of the characters in the picture is: happy.”). Each participant completed 36 trials in total. The eye movement data were recorded during the task for offline analysis. In order to ensure that each participant understood the emotion recognition task, one trial was conducted as practice before the actual experiment began (Figure 2).

#### 2.3.3. Data Processing

Fixations were defined by the number of consecutive gaze points. A fixation threshold of 80 ms was applied to avoid unconscious looking. Only gaze points where both eyes had been successfully tracked by the eye tracker were used. We divided the images into three areas of interest (AOI): the eyes, mouth+nose, and the rest of the face (rest). The eye-AOI covered 3% of the image area, as did the mouth+nose-AOI; the rest-AOI covered 94% of the image. The first phase of the analysis was to automatically identify nonfixation data, including blinks, glances, head motion, and poor calibration, by identifying fast eye motion and gazes away from the presentation screen. Trials with no fixations on the picture were excluded. We measured the percentage (%) of visual fixation time on the three AOIs and the accuracy of emotion judgment.

### 2.4. Neuronavigated rTMS

TMS was performed using a Magstim Rapid^2^ stimulator (Magstim Company, Whitland, UK). A typical iTBS session lasted 190 s and consisted of a burst of three pulses delivered at 50 Hz (70% resting motor threshold (RMT)) for two seconds, which was repeated every 8 s for a total of 600 pulses. In this study, three iTBS sessions were delivered with a 15-minute intersession interval using a 70 mm air-cooled figure-of-eight coil. High-resolution anatomical images were acquired (field of view = 256 × 256 mm, slice thickness/gap = 1/0 mm, in-plane resolution = 256 × 256, repetition time = 8.15 ms, echo time = 3.18 ms, flip angle = 8 degrees, and 176 sagittal slices) for neuronavigation. The stimulus target, the rpSTS, was defined as a sphere with a center radius of 6 mm based on the Montreal Neurological Institute (MNI) coordinates [55, -53, 17], [34]. Using the inverse matrix generated by T1 segmentation in SPM12 (http://www.fil.ion.ucl.ac.uk/SPM) and TMStarget software (http://www.brainhealthy.net) [35], the target was transformed into T1 space for each participant [35]. Then, each individual's target was imported into a frameless neuronavigation system (Visor2.0, Advanced Neuro Technologies, Hengelo, Netherlands).

Pulses were delivered at 70% of the RMT [36]. In order to measure the RMT, motor-evoked potential amplitudes of the abductor pollicis brevis muscle were recorded using Ag/AgCl surface electrodes when the left “hand knob” area was stimulated. The electromyography (EMG) signal was amplified, digitized, and displayed on a computer screen by the Rogue EMG device. RMT was defined as the minimum intensity that caused a small response (>50 mV) more than five times in 10 consecutive trials and was measured prior to each rTMS [23, 35]. The sham stimulus was delivered by a placebo coil (Magstim Company), which produced a similar feeling on the participant's scalp as the real coil but did not induce an efficient current in the cortex. The coil was held tangentially to the right skull, with the long axis parallel to the uncinate gyrus of the target (pSTS) and the center over the target sphere (Figure 1).

### 2.5. Functional Magnetic Resonance Imaging

#### 2.5.1. Data Acquisition

MRI scans of all subjects were performed at the MRI center of the medical center of the University of Science and Technology of China. MRI datasets were obtained with the aid of a 3-T scanner (Discovery 750; GE Healthcare, Milwaukee, WI). Foam padding and earplugs were applied to minimize head motion and scanner noise for all subjects. During resting-state fMRI (rsfMRI) scanning, subjects were instructed to rest with their eyes closed without falling asleep. Functional images were acquired using an echo planar imaging sequence (repetition/echo time = 2400/30 ms, flip angle = 90 degrees, and 217 volumes). A total of 46 transverse slices (field of view, 192 × 192 mm^2^; 64 × 64 in-plane matrices; section thickness without intersection gap, 3 mm; and voxel size, 3 × 3 × 3 mm^3^) were acquired parallel to the anteroposterior commissure line. iTBS was performed in a separate room beside the scanner.

#### 2.5.2. Data Processing

Functional image processing was carried out using the DPARSF (http://rfmri.org) [37], SPM12 (http://www.fil.ion.ucl.ac.uk/spm) toolkits, REST (http://www.restfmri.net/) [38], and TMStarget (http://www.brainhealthy.net/). The preprocessing included (1) deleting the first five volumes; (2) slice timing and realignment; (3) coregistering T1 to functional images; (4) normalizing T1 to the MNI space and segmenting it into gray matter, white matter, and cerebrospinal fluid (spatial resolution: 3 × 3 × 3); (5) smoothing images with a 4 mm isotropic Gaussian kernel; and (6) filtering the temporal bandpass (0.01–0.1 Hz) and regressing out 27 nuisance signals (global mean, white matter, cerebrospinal fluid signals, and 24 head motion parameters). Subsequently, Pearson's correlations were conducted between the time series of the target regions-of-interest (ROI) and that of each voxel in the whole brain and normalized using Fisher's *z* transformation. The target ROI was defined as a sphere with a 6 mm radius centered at MNI coordinates [55, -53, 17]. No subject had head motion exceeding 3 mm of translation or three degrees of rotation during the fMRI acquisition.

### 2.6. Statistical Analysis

For the baseline measures, the Shapiro-Wilk test was conducted to check whether the measurement data were normally distributed. *χ*^2^ or two-sample *t*-tests were used to compare the differences in baseline in the two groups. For the eye tracking measures, two-way (group by time) repeated-measure ANOVA was used to analyze the changes in fixation time (%) of the three AOIs and emotion recognition accuracy. All post hoc *P* values were adjusted using Bonferroni's multiple comparison correction. In our situation, the raw *P* values from post hoc analysis were multiplied by 2. The effect size of post hoc analysis was computed using Cohen's *d*.

To identify regions significantly correlated with the target, one-sample *t*-tests were performed for the target-to-whole brain functional connectivity maps for each condition (e.g., pre- and post-rTMS condition). Then, voxels surviving in either condition (uncorrected voxel level *P* < 0.05) constituted a mask for comparing the functional alterations after rTMS using two-way repeated-measure ANOVA. This comparison was performed through a toolbox in SPM12, Statistic non-Parameter Mapping (SnPM) [39]. To control the family-wise error (FWE) in multiple comparisons, we first set a voxel level threshold of *P* < 0.01. Then, only clusters larger than a given volume were reported as having survived the cluster-level correction, *P*_corr_ < 0.05. Regions surviving the FEW corrections were set as ROI for further analysis. The signals of ROI were represented by the average signal within spheres centered at the peak value of clusters with a 6 mm radius.

Pearson correlation analysis was performed between the altered rsFC in ROI and behavior measures (e.g., fixation time on the eye-AOI and emotion recognition accuracy) in the real group. Significance was determined at *P* < 0.05 (two tailed).

## 3. Results

### 3.1. Background Measures

To ensure the quality of the data, the data (average across trials) exceeding three standard deviations from the mean were defined as outliers. Subjects with one outlier in either fixation time or emotion recognition accuracy were excluded from all analyses. As a result, three participants in the real group were excluded. Additionally, two participants were excluded due to unqualified calibration (>1°) during eye tracking. Finally, 12 and 15 participants were included in the real and sham groups for analysis, respectively. There was no significant difference in gender, age, RMT, and other neuropsychological tests between the two groups (*P* > 0.05, Table 1). In total, 56% of the participants in the real group (7 of 12) and 31% in the sham group (4 of 15) guessed correctly to which group they belonged (Fisher's exact test, *P* = 0.10). When outliers were included, no significant difference was found in the baseline comparisons of the data (see Supplementary Materials).

### 3.2. Emotion Recognition Task

**Figure 3:**
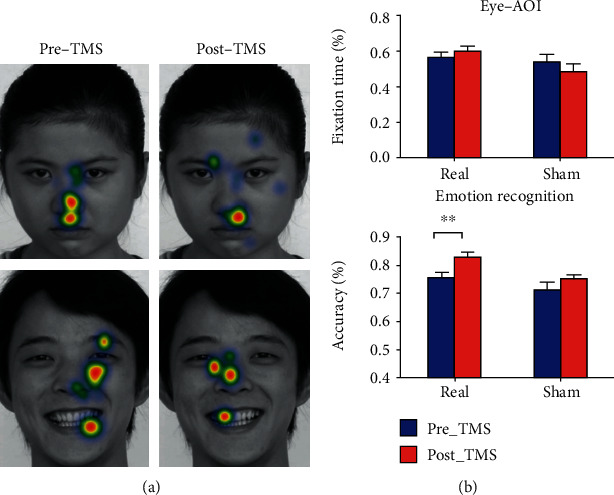
Behavioral changes after intervention. Heatmaps of one participant illustrate the eye fixation pattern before and after real intervention (a). Warm and cold colors denote a greater and fewer number of fixations, respectively. Bar graph illustrating the fixation time on eye-AOI and emotion recognition accuracy in the real and sham groups, before and after the intervention (b). The emotion recognition accuracy was significantly improved after intervention in the real group, but not the sham group. Error bars indicate SEM. ^∗∗^*P* < 0.01.

For the fixation time (%) of the eye-AOI (Figures 3(a) and 3(b)), no significant (group by time) interaction (*F*_[1, 25]_ = 2.14, *P* = 0.16), group (*F*_[1, 25]_ = 2.00, *P* = 0.17), or time (*F*_[1, 25]_ = 0.12, *P* = 0.73) effect was found. No significant effect was found for the other AOIs (see details in the Supplementary Materials). For emotion recognition accuracy, the (group by time) interaction was not significant (*F*_[1, 25]_ = 1.36, *P* = 0.25), but the time (*F*_[1, 25]_ = 14.04, *P* = 0.0009) and group (*F*_[1, 25]_ = 4.87, *P* = 0.04) main effects were significant. Although the interaction effect was not significant, the accuracy of emotion recognition was significantly improved in the real group (*t* = 3.30, *P* = 0.006, *d* = 0.90), but not the sham group (*t* = 1.93, *P* = 0.12, *d* = 0.53; Figure 3(b)). Power analysis in GPower 3.1 showed a relative low power (=0.77) for the interaction effect (effect size *f* = 0.23).

### 3.3. Resting-State Functional Connectivity

**Figure 4:**
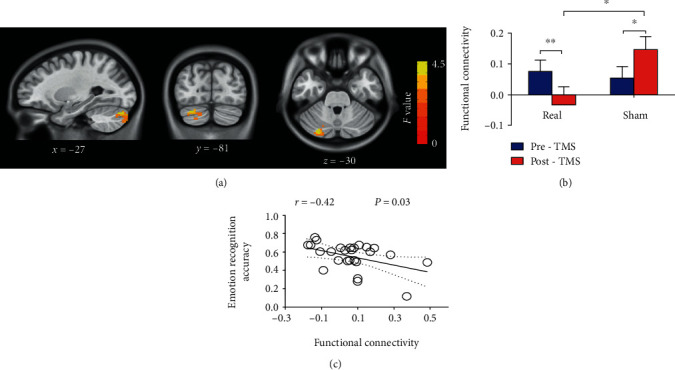
Resting-state functional connectivity (rsFC) alterations after intervention. The cerebellum showing a significant (group by time) interaction in rsFC (a). A sphere area from the significant cerebellum cluster was defined as the region-of-interest (ROI). The bar graph showing rsFC values (mean and SD) of the cerebellum ROI in each group (b). The rsFC in the ROI decreased in the real group and increased in the sham group after intervention. The real group showed lower rsFC after intervention than the sham group. Before intervention, rsFC of the cerebellum ROI was negatively correlated with emotion recognition accuracy (c). Error bars indicate SEM. ^∗^*P* < 0.05, ^∗∗^*P* < 0.01.

All participants (*n* = 32) were included in the imaging analyses. Paired *t*-tests (post- vs. pre-rTMS) on functional connectivity maps were first performed for both groups independently (voxel *P* < 0.05). Two-way repeated-measure ANOVA indicated a significant cluster in the left cerebellum (Figure 4(a), Table S2), which was then set as a ROI for further analysis. rsFC in the ROI showed a significant (group by time) interaction effect (*F*_[1, 30]_ = 17.86, *P* = 0.0002, Figure 4(b)). Post hoc analyses indicated that rsFC in the ROI decreased in the real group (*t* = 3.23, *P* = 0.006, *d* = 0.71) and increased in the sham group after intervention (*t* = 2.74, *P* = 0.02, *d* = 0.84). At the end of the intervention, the real group showed smaller rsFC than the sham group (*t* = 2.81, *P* = 0.01, *d* = 0.61). All post hoc *P* values were adjusted using Bonferroni's correction. There was no significant difference between the groups before the intervention (*t* = 0.34, *P* > 0.99, Figure 4(b)).

Ten subjects in the real group and 12 subjects in the sham group completed the follow-up visit. To track the dynamic alteration of the cerebellar ROI, the follow-up data were compared with the pre- and post-rTMS data using paired *t*-tests (Table S2). After Bonferroni's correction, the ROI rsFC in follow-up was similar to the pre-rTMS state (*t* = 1.94, *P* = 0.16, *d* = 0.77) and higher than the post-rTMS state (*t* = 3.11, *P* = 0.02, *d* = 0.98) in the real group; no significant difference between the states was found for the sham group (follow-up vs. pre-rTMS, *t* = 2.45, *P* = 0.06, *d* = 0.67; follow-up vs. post-rTMS, *t* = 1.11, *P* = 0.60, *d* = 0.32).

### 3.4. Behavior Imaging Correlation

The baseline rsFC values between the rpSTS and the cerebellum showed a significant negative correlation with the baseline emotion recognition accuracy (*r* = −0.42, *P* = 0.03; Figure 4(c)) and a marginal significant correlation with the baseline fixation time (%) on eyes (*r* = −0.38, *P* = 0.05). The correlation analyses for other AOIs are shown in the Supplementary Materials. No significant correlation was found between the altered rsFC (rpSTS and cerebellum) and altered behavior measures in the fixation time on the eye-AOI (*r* = −0.15, *P* = 0.45) or emotion recognition accuracy (*r* = −0.09, *P* = 0.64). The correlation graphs are shown in the Supplementary Materials.

### 3.5. Adverse Effects

Five participants felt transient discomfort during stimulations because of muscle twitches around the eyes. No other adverse effects, such as headaches or seizures, were reported. Self-reports indicated that iTBS was well tolerated (average discomfort rating = 0.89 ± 1.04, with the possible range of 0-10, where higher values indicate more discomfort).

## 4. Discussion

Using a randomized and sham control design, we preliminarily investigated the effect of iTBS on ALT individuals. We mainly observed two aspects. First, we found that five consecutive days of iTBS increased the accuracy of facial emotion recognition, implicating an improved ability to understand facial expressions. Second, the rsFC between the rpSTS and the left cerebellar Crus I/II significantly decreased in the real group after intervention. Prior to intervention, the rsFC in the left cerebellum was negatively correlated with emotion recognition accuracy. In summary, iTBS has the potential to improve emotion perception of ALT individuals and regulate cortical network function.

### 4.1. Emotion Recognition Task

Accurate reading of other people's facial expressions is a key factor for successful social interaction [40]. As predicted, our results showed that excitatory rTMS of the rpSTS (real group) increased the emotion recognition accuracy of ALT individuals. In addition, gaze time on eyes did not change significantly after real stimulations. These data suggest that the improvement of accuracy was not caused by longer attention to eyes. However, the accuracy increase in the real group was not significantly larger than that in the sham group. By increasing sample size, future studies with higher statistical power may be able to dissociate the rTMS effect from the placebo effect.

The human pSTS is a core node of a network for face perception [41, 42]. For instance, neuroimaging studies have shown that the pSTS was activated by static images of faces [43]. By producing virtual lesions on the rpSTS, online TMS (10 Hz for 500 ms) significantly impaired performance of ALT individuals on facial expression recognition tasks [18]. Besides, the rpSTS function is also related to gaze perception [44]. Saitovitch et al. found that inhibitory rTMS of the rpSTS induced fewer fixations to the eyes [28]. Motivated by their work, we selected excitatory rTMS in this study. However, we did not observe an increased fixation time on the eyes after real stimulations. This is probably because normalizing gaze behavior is much harder than disrupting it. In ASD, fMRI studies have also demonstrated a link between neurologic abnormalities in the pSTS and emotional perception deficits. Overall, we speculate that rpSTS regulation by rTMS may be a potential therapy for improving emotional recognition in ASD.

### 4.2. Functional Connectivity

To investigate the neural correlations underlying the behavior effect, we used functional MRI to analyze rsFC alterations after intervention. We found that the rsFC between the left cerebellar Crus I/II and the rpSTS significantly decreased in the real group after iTBS. This was consistent with previous studies indicating that rTMS was able to affect brain resting-state connectivity [23, 35, 45]. Follow-up results showed that after more than two months, the FC in the left cerebellum slowly returned to baseline. Thus, the change of this neural circuit caused by iTBS is temporary and can return to normal within a certain period of time.

Our behavior imaging correlation analysis suggested that the rsFC of the cerebellum was related to the emotional recognition ability in ALT individuals. For instance, fMRI studies showed that the posterior cerebellum (especially the Crus I/II) participates in emotional and social processing through the corticocerebellar connection to the pSTS [46, 47]. Further, a TMS study found that excitatory stimulation of the left cerebellum reduced the accuracy of processing facial emotions [48]. The cerebellum forms multiple circuits with cortical regions, which forms the basis for motor, language, and social processing [49]. Through these circuits, cerebellar dysfunction could affect the core symptoms (social deficits) of ASD. The previous literature has found decreased Purkinje cells [50], abnormal structure [51, 52], function [53], and aberrant rsFC on the left cerebellum in ASD [54, 55]. Those studies revealed that rTMS targeting the rpSTS may provide a new treatment method for modulating cerebral-cerebellar circuits and their function in ASD.

There were also some limitations in this study. First, this was a small-sample study. The robustness and reproducibility of our findings need to be further validated. With a larger sample size, the marginal significant correlation between the cerebellum rsFC and the baseline fixation time may become prominent. Second, the stimulation period was too short. For instance, the guidelines for depression treatment suggest six to eight weeks of stimulation [56]. To induce a more prominent effect, future research should use longer intervention periods or higher stimulation frequencies (e.g., multisessions/day). Third, our behavior and imaging experiments were conducted separately. This may be one of the reasons that we failed to observe a significant correlation between behavior and imaging changes. Fourth, autism subjects are predominantly males [57, 58]. But there were fewer male subjects in this study than females, which may have influenced the generalization of the findings. It would be interesting to investigate the interaction between genders on rTMS modulation in future studies with larger sample sizes. Finally, it would be better if the neuropsychological tests could be estimated at the end of rTMS. It is important to explain whether the improvements in social cognition were specific or just a reflection of a general improvement in performance of all the applied tasks.

## 5. Conclusion

This study preliminarily explored the effect of excitatory rTMS of the rpSTS on emotional recognition in ALT individuals. In the real group, emotional recognition was improved, and the rsFC between the rpSTS and the left cerebellum was significantly decreased following five consecutive days of iTBS. These findings suggest that iTBS of the rpSTS could improve emotion perception in ALT individuals by modulating the associated neural networks. In the future, it would be valuable to test this stimulation protocol in ASD patients for therapeutic purposes.

## Figures and Tables

**Table 1:** Baseline characteristics of the participants.

Variable	iTBS	Placebo	*t*/*χ*^2^	*P* value
Demographic				
Gender (M/F)	6/6	6/9	0.52	0.60
Age (years)	22.27 ± 0.45	22.33 ± 0.91	-0.20	0.84
AQ	130.05 ± 3.37	133.10 ± 3.32	-1.22	0.23
RMT	58.20 ± 6.23	58.67 ± 5.21	-0.22	0.83
Neuropsychological				
HAMA	5.67 ± 0.65	5.93 ± 0.48	-0.86	0.40
HAMD	3.47 ± 1.48	3.60 ± 0.79	-0.42	0.68
MoCA	29.33 ± 0.37	29.47 ± 0.51	-0.73	0.47
Digit span (forward)	9.73 ± 0.32	9.73 ± 0.62	0	>1
Digit span (backward)	6.93 ± 0.61	6.93 ± 0.56	0	>1
Stroop color test	11.34 ± 1.63	10.67 ± 1.41	1.51	0.14
Stroop word test	11.64 ± 1.31	10.80 ± 1.32	1.79	0.08
Stroop interference test	18.90 ± 1.62	18.65 ± 1.51	0.40	0.70
Trail making A	27.03 ± 1.79	27.08 ± 1.69	-0.07	0.94
Trail making B	49.96 ± 2.78	49.14 ± 3.62	0.67	0.51

AQ: autism spectrum quotient; F: female; HAMA: Hamilton Anxiety Rating Scale; HAMD: Hamilton Depression Rating Scale; M: male; MoCA: Montreal Cognitive Assessment Test; iTBS: intermittent theta burst stimulus; RMT: resting motor threshold.

## Data Availability

The data used to support the findings of this study are available from the corresponding author upon request.

## Supplementary Materials

Supplementary Materials Supplementary analysis and findings.
