# Supplementary material for: Increased Accuracy of Emotion Recognition in Individuals with Autism-Like Traits after Five Days of Magnetic Stimulations
Source: Neural Plast. 2020 Jul 4;2020:9857987. doi: 10.1155/2020/9857987 (PMC7355343; doi:10.1155/2020/9857987)
Supplement: Supplementary Materials — Supplementary analysis and findings. [file 9857987.f1.doc]

**Supplementary for**

**Increased accuracy of emotion** **recognition in individuals with autism-like traits after five days of magnetic stimulations**

Pingping Liu*, Guixian Xiao*, Kongliang He*, Long Zhang, Xinqi Wu, Dandan Li, Chunyan Zhu, Yanghua Tian, Panpan Hu, Bensheng Qiu, Gong-Jun JI^#^, Kai Wang^#^

1. **Other results of the analysis in main text**

**Emotion Recognition Task**

In two-way ANOVA, no significant interaction effect (group × time) was found for the fixation time (%) of the (mouth+nose)-AOI (F_[1, 25]_ = 0.01, *P* = 0.91) or the rest-AOI (F _[1, 25]_ = 3.42, *P* = 0.08). No significant main effect was found for group factors in the (mouth+nose)-AOI (F _[1, 25]_ = 0.74, *P* = 0.40) and the rest-AOI (F _[1, 25]_ = 1.20, *P* = 0.28), or time factors in the (mouth+nose)-AOI (F _[1, 25]_ = 0.61, *P* = 0.44) and the rest-AOI (F _[1, 25]_ = 1.24, *P* = 0.28) (Fig.S1, table S1).

**
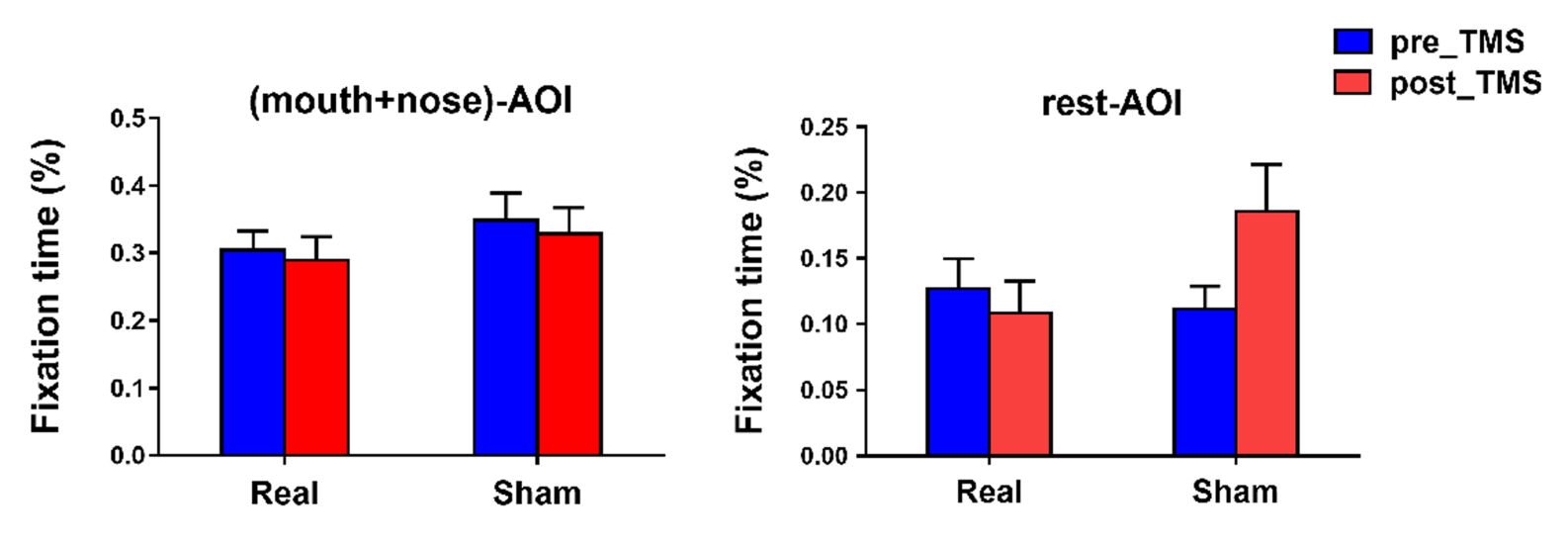
**

**Figure S1** Behavioral changes after intervention. Bar graph illustrate the fixation time on the (mouth+nose) and rest AOI in the real and sham group, before and after the intervention.

**Behavior-imaging Correlation**

The rsFC value between rpSTS and cerebellum showed a significant positive correlation with the fixation time (%) on the rest-AOI at baseline (r = 0.54, *P* = 0.004; Fig. S2A), but we observed no significant correlation with the fixation time (%) on the (mouth+nose)-AOI (r = 0.24, *P* = 0.22; Fig. S2B) or eye-AOI (r = −0.38, *P* = 0.05; Fig. S2C). The positive correlation on the rest-AOI indicates that facial triangle is negatively correlated with rsFC value at baseline.


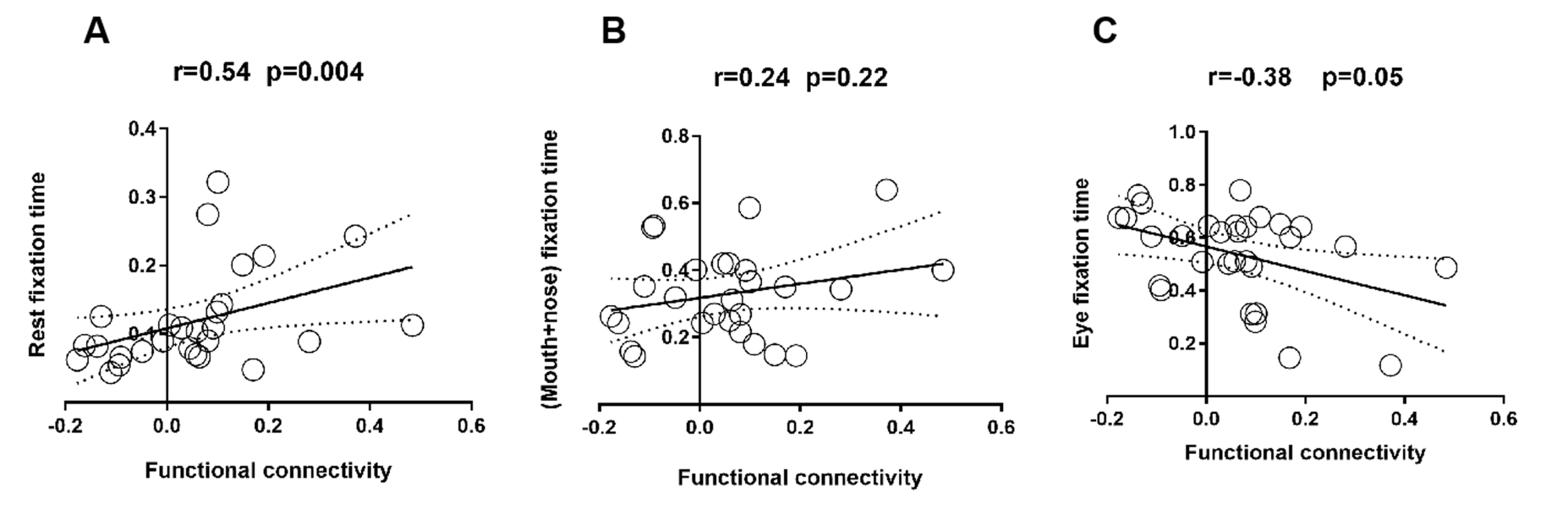


**Figure S2** Behavior-imaging Correlation. Before intervention, rsFC of the cerebellum ROI positively correlated with the fixation time (%) on rest-AOI (A), but no significant correlation in the fixation time (%) on (mouth+nose)-AOI (B) or eye-AOI (C).

After intervention, no significant correlation was found between the altered rsFC (in cerebellum) and altered behavior measures in emotion recognition accuracy (*r* = -0.09, *P* = 0.64; Fig. S3A) or in the fixation time on the eye-AOI (*r* = -0.15, *P* = 0.45; Fig. S3B), on the (mouth+nose)-AOI (r =-0.33, *P* = 0.09; Fig. S3C) and on the rest-AOI (r = 0.36, *P* = 0.06; Fig. S3D).


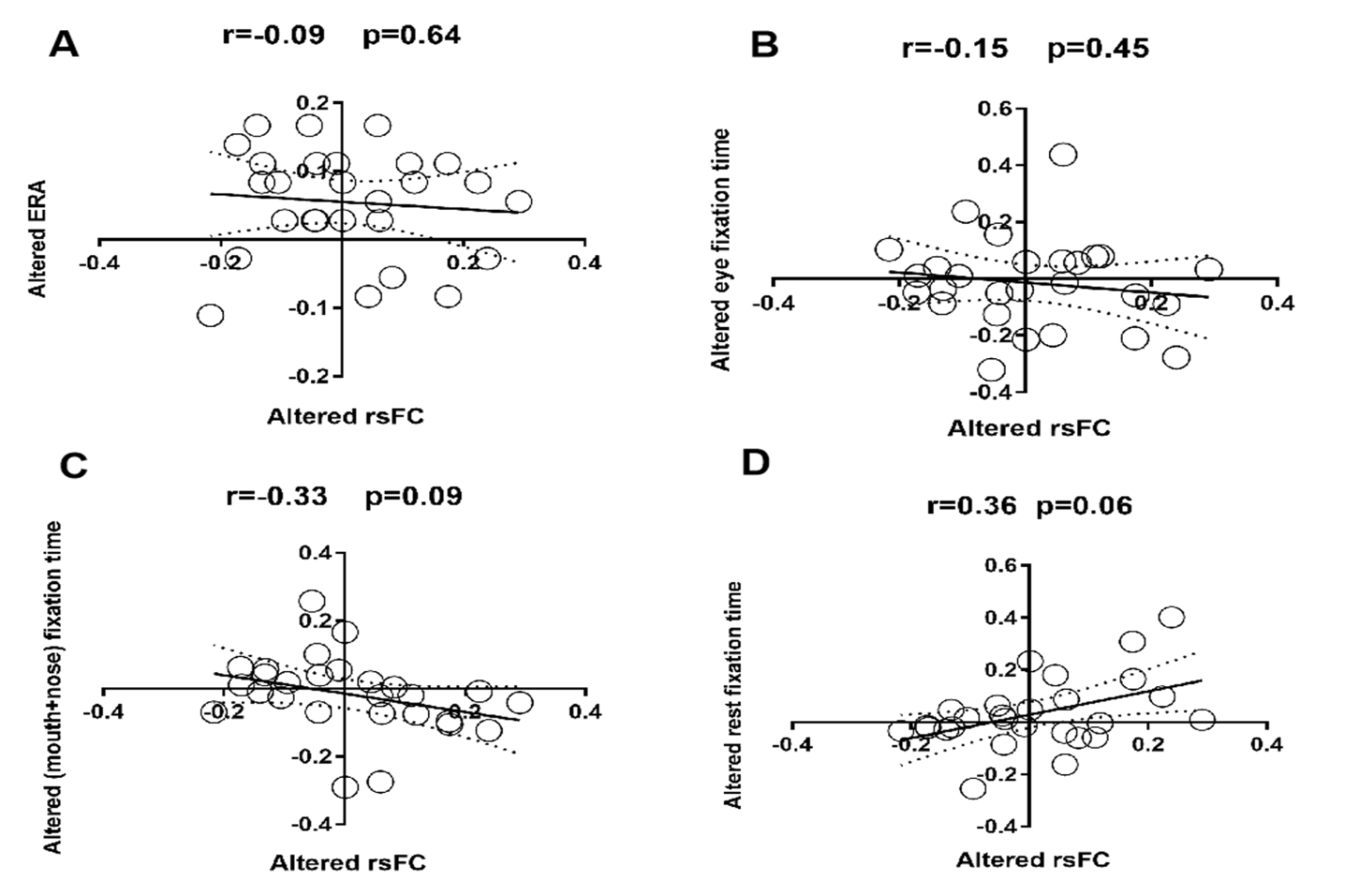


**Figure S3** Correlation between rsFC and fixation time changes after intervention. The altered rsFC (in cerebellum cluster) and altered behavior measures in the emotion recognition accuracy or the fixation time on the three AOIs was not significant.

**Table S1. Emotion recognition task**

| **Measures** | **Real (n=12)** | | **Sham (n=15)** | |
| --- | --- | --- | --- | --- |
|  | pre-TMS | post-TMS | pre-TMS | post-TMS |
| **Fixation time (%)** |  |  |  |  |
| eye-AOI | 0.57±0.11 | 0.60±0.10 | 0.54±0.17 | 0.49±0.19 |
| (mouth+nose)-AOI | 0.30±0.10 | 0.29±0.12 | 0.35±0.16 | 0.33±0.15 |
| rest-AOI | 0.13±0.08 | 0.11±0.08 | 0.11±0.07 | 0.19±0.14 |
| **Emotion recognition accuracy (%)** | 0.75±0.07 | 0.83±0.08 | 0.72±0.11 | 0.76±0.06 |

Table S2 Resting-state Functional Connectivity

| **Functional Connectivity** | **pre-TMS** | **post-TMS** | **follow-up** |
| --- | --- | --- | --- |
| **Real** | 0.08±0.15 | -0.03±0.23 | 0.17±0.21 |
| **Sham** | 0.05±0.15 | 0.15±0.17 | 0.11±0.15 |

1. **Supplementary analysis and findings**

In the main text, data of three participants were identified as the extremums and excluded from analysis. As a supplementary analysis, we included these 3 participants here. There was no significant difference in gender, age, RMT and other neuropsychological tests between the two groups (*P* > 0.05, table S3).

Table S3. Baseline measures of participants

| **Variable** | **iTBS** | **Placebo** | | **t/χ^2^** | | ***p*-Value** |  |
| --- | --- | --- | --- | --- | --- | --- | --- |
| **Demographic** |  |  | |  |  | |  |
| Gender (M/F) | 6 / 9 | | 6 / 9 | 0 | 1 | |  |
| Age (years) | 22.25±0.75 | | 22.35±0.91 | -0.23 | 0.82 | |  |
| AQ | 130.00±3.27 | | 133.10±3.32 | -1.30 | 0.21 | |  |
| RMT | 58.78±6.11 | | 58.67±5.21 | 0.04 | 0.97 | |  |
| **Neuropsychological** |  | |  |  |  | |  |
| HAMA | 5.75±0.73 | | 5.93±0.48 | -0.54 | 0.59 | |  |
| HAMD | 3.58±0.41 | | 3.60±0.79 | -0.05 | 0.96 | |  |
| MoCA | 29.33±0.43 | | 29.47±0.51 | -0.68 | 0.50 | |  |
| Digit span (forward) | 9.83±0.65 | | 9.73±0.62 | 0.36 | 0.72 | |  |
| Digit span (backward) | 6.92±0.73 | | 6.93±0.56 | -0.06 | 0.95 | |  |
| Stroop Color test | 11.35±1.92 | | 10.67±1.41 | 1.37 | 0.18 | |  |
| Stroop Word test | 11.61±1.35 | | 10.80±1.32 | 1.61 | 0.12 | |  |
| Stroop Interference test | 18.83±1.57 | | 18.65±1.51 | 0.25 | 0.80 | |  |
| Trail Making A | 27.07±1.82 | | 27.08±1.69 | -0.02 | 0.98 | |  |
| Trail Making B | 49.82±1.70 | | 49.14±3.62 | 0.51 | 0.62 | |  |
| AQ: Autism spectrum questionnaire, F: female, HAMA: Hamilton Anxiety Rating Scale, HAMD: Hamilton Depression Rating Scale, iTBS: intermittent Theta-burst stimulus, M: male, MoCA: Montreal Cognitive Assessment Test, RMT: Resting motor threshold | | | | | | | |

**Emotion recognition task**

In two-way ANOVA, no significant interaction effect (group × time) was found for the fixation time (%) of the eye-AOI (F _[1, 28]_ = 2.37, *P* = 0.14)，the (mouth+nose)-AOI (F_[1, 28]_ = 0.03, *P* = 0.86) or the rest-AOI (F _[1, 28]_ = 3.61, *P* = 0.07) (Fig. S4A, table S4). No significant effect was found for group factor in the eye-AOI (F _[1, 28]_ = 0.50, *P* = 0.48), (mouth+nose)-AOI (F _[1, 28]_ = 0.52, *P* = 0.48) or the rest-AOI (F _[1, 28]_ = 0.0042, *P* = 0.99). No significant effect was found for group factor time factor in the eye-AOI (F _[1, 28]_ = 0.16, *P* = 0.70), (mouth+nose)-AOI (F _[1, 28]_ = 0.62, *P* = 0.44) or the rest-AOI (F _[1, 28]_ = 1.23, *P* = 0.28). For emotion recognition accuracy (Table S4), group by time interaction (F _[1,28]_ = 2.40, *P* = 0.13) and group main effects (F _[1,28]_ =2.69, *P* = 0.11) were not significant, while the time effect was significant (F _[1,28]_ = 11.96, *P* = 0.0018). Post hoc analyses indicated that the accuracy was significantly improved in the real group (t = 3.54, *P* = 0.0028, d=0.73), but not in the sham group (t = 1.35, *P* = 0.34, d=0.53) (Fig. S4B).


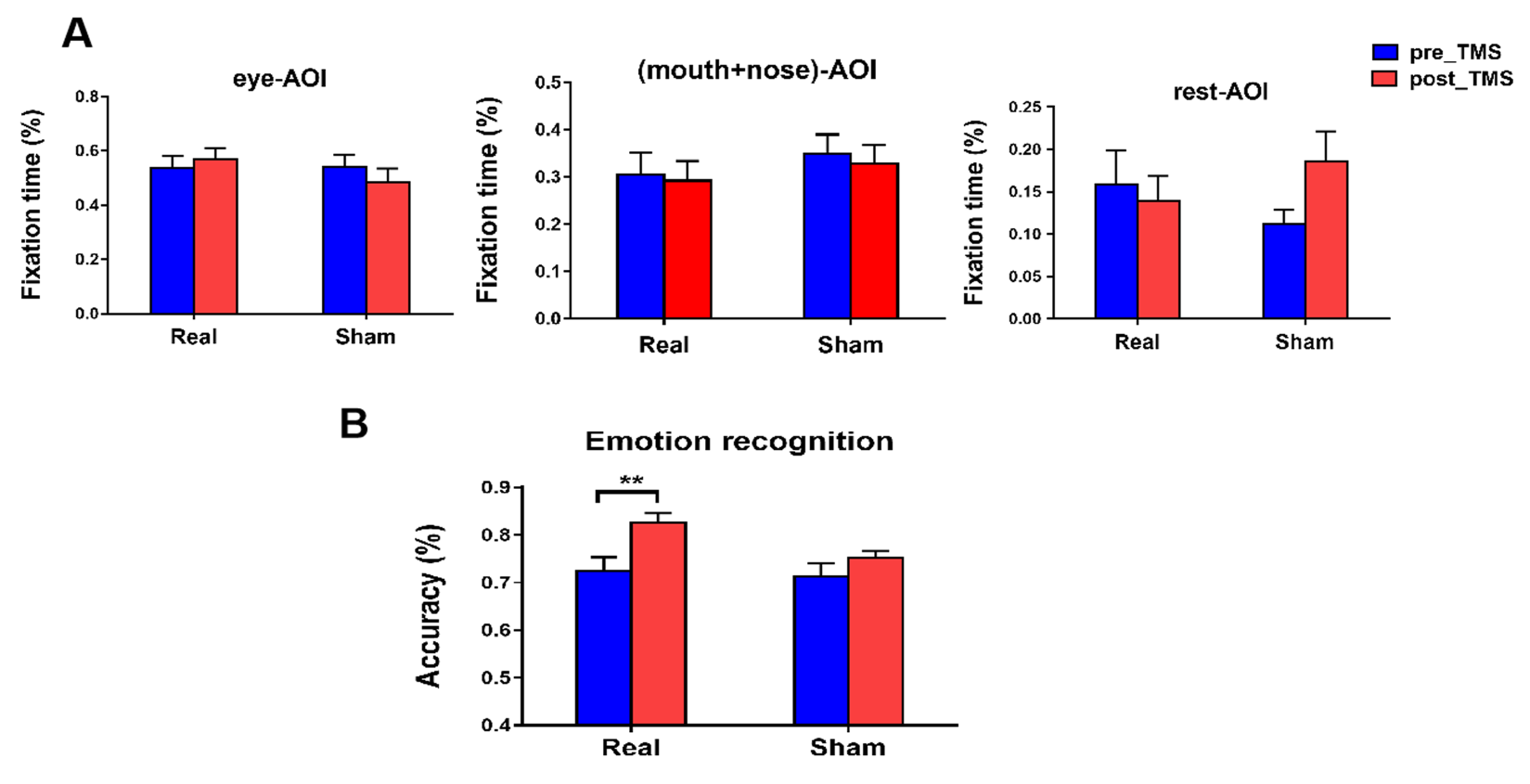


**Figure S4** Behavioral changes after intervention without removing the extremum. Bar graph illustrate the fixation time on three AOIs (A) and emotion recognition accuracy (B) in the real and sham group, before and after the intervention. The emotion recognition accuracy was significantly improved after intervention in the real group, but not the sham group. Error bars indicate SEM. ***P* < 0.01.

Table S4. Emotion recognition task

| **Emotion recognition task** | **Real (n=15)** | | **Sham (n=15)** | |
| --- | --- | --- | --- | --- |
|  | pre-TMS | post-TMS | pre-TMS | post-TMS |
| **Fixation time (%)** |  |  |  |  |
| eye-AOI | 0.54±0.17 | 0.57±0.16 | 0.54±0.17 | 0.49±0.19 |
| (mouth+nose)-AOI | 0.30±0.18 | 0.29±0.16 | 0.35±0.16 | 0.33±0.15 |
| rest-AOI | 0.16±0.16 | 0.14±0.11 | 0.11±0.07 | 0.19±0.14 |
| **Emotion recognition accuracy (%)** | 0.72±0.11 | 0.83±0.08 | 0.72±0.11 | 0.76±0.06 |

**Behavior-imaging Correlation**

The rsFC value between rpSTS and cerebellum showed a significant negative correlation with the emotion recognition accuracy at baseline (r=−0.47, *P*=0.008; Fig. S5A) and fixation time (%) on the eye-AOI(r=−0.40,*P* =0.03;Fig.S5B), and a significant positive correlation with the rest-AOI at baseline (r = 0.45, *P* = 0.01; Fig.S5C). But we observed no significant correlation with the fixation time (%) on the (mouth+nose)-AOI (r = 0.23, *P* = 0.21; Fig. S5D).


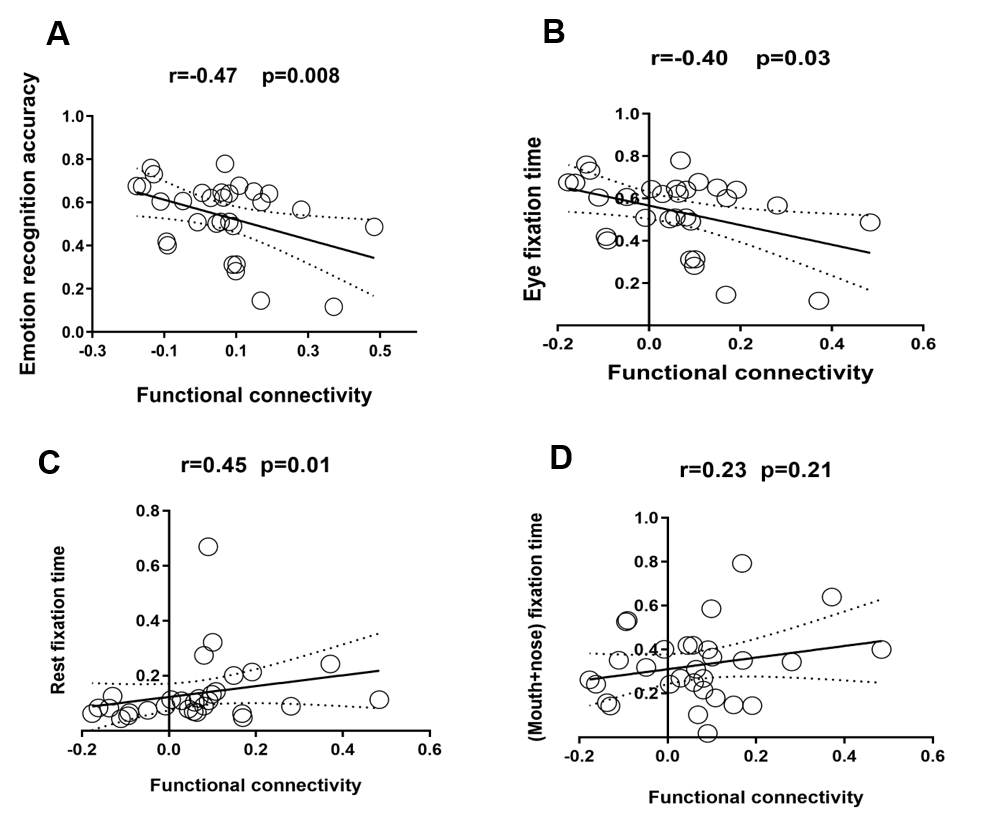


**Figure S5** Behavior-imaging Correlation at baseline. Before intervention, rsFC of the cerebellum ROI negatively correlated with the emotion recognition accuracy (A) and the fixation time (%) on eyes (B), and positively correlated with the fixation time (%) on rest-AOI (C)

After intervention, no significant correlation was found between the altered rsFC (in cerebellum) and altered behavior measures in emotion recognition accuracy (*r* = -017, *P* = 0.35; Fig. S6A) and in the fixation time on the eye-AOI (*r* = -0.04, *P* = 0.83; Fig. S6B), the rest-AOI (r =-0.30, *P* = 0.10; Fig. S6C) or the (mouth+nose)-AOI at baseline (r = 0.33, *P* = 0.08; Fig. S6D).


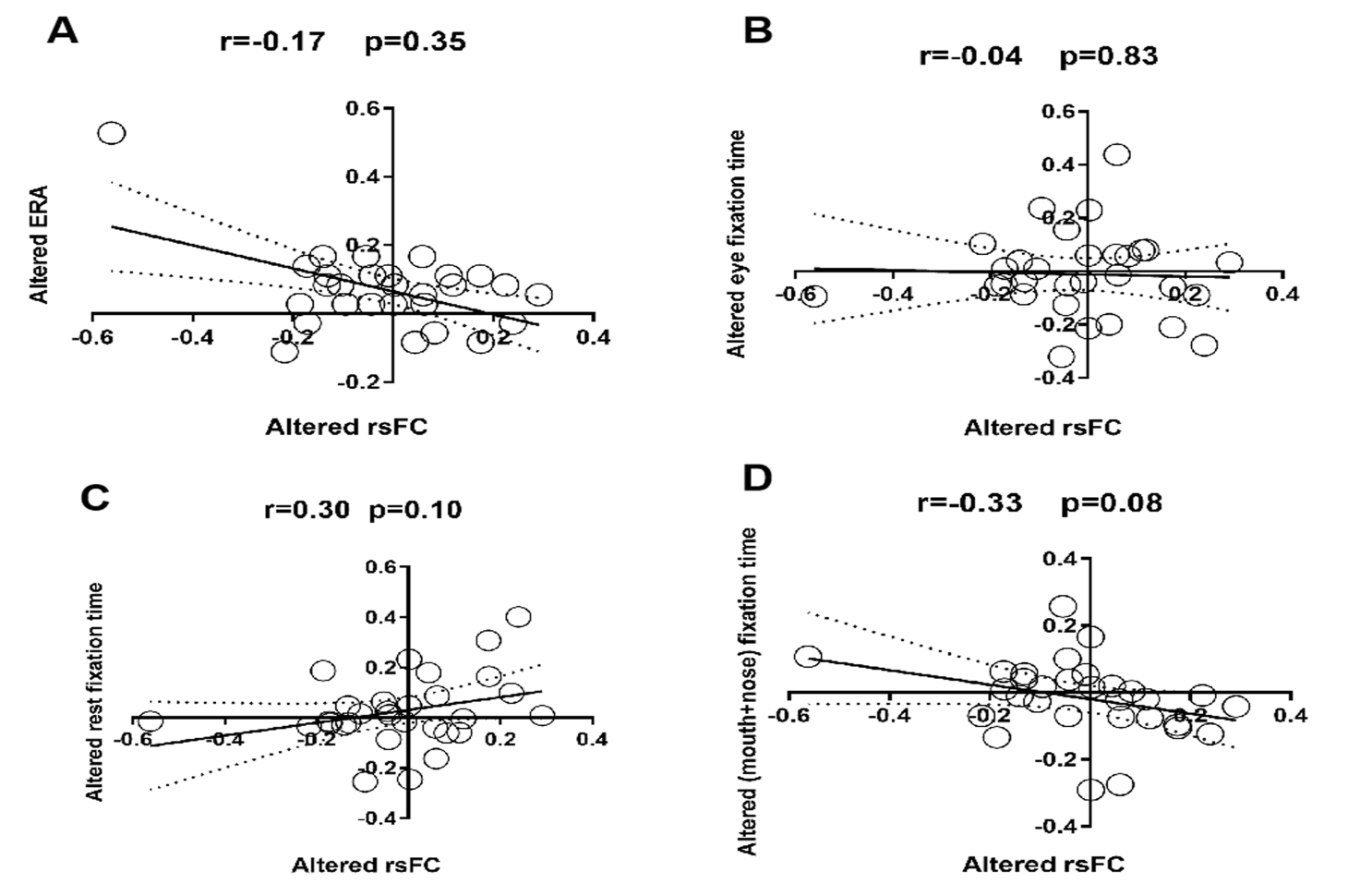


**Figure S6** Correlation between rsFC and fixation time changes after intervention. The altered rsFC (in cerebellum cluster) and altered behavior measures in the emotion recognition accuracy or the fixation time on the three AOIs was not significant.
